# Pepino mosaic virus antagonizes plant m^6^A modification by promoting the autophagic degradation of the m^6^A writer HAKAI

**DOI:** 10.1007/s42994-023-00097-6

**Published:** 2023-02-23

**Authors:** Hao He, Linhao Ge, Zhaolei Li, Xueping Zhou, Fangfang Li

**Affiliations:** 1 State Key Laboratory for Biology of Plant Diseases and Insect Pests, Institute of Plant Protection, Chinese Academy of Agricultural Sciences, Beijing, 100193 China; 2 State Key Laboratory of Rice Biology, Institute of Biotechnology, Zhejiang University, Hangzhou, 310058 China

**Keywords:** Pepino mosaic virus, HAKAI, Autophagy, Beclin1

## Abstract

Autophagy plays an active anti-viral role in plants. Increasing evidence suggests that viruses can inhibit or manipulate autophagy, thereby winning the arms race between plants and viruses. Here, we demonstrate that overexpression of an m^6^A writer from *Solanum lycopersicum*, SlHAKAI, could negatively regulate pepino mosaic virus (PepMV) infection, inhibit viral RNA and protein accumulations by affecting viral m^6^A levels in tomato plants and vice versa. The PepMV-encoded RNA-dependent RNA polymerase (RdRP) directly interacts with SlHAKAI and reduces its protein accumulation. The RdRP-mediated decreased protein accumulation of SlHAKAI is sensitive to the autophagy inhibitor 3-methyladenine and is compromised by knocking down a core autophagy gene. Furthermore, PepMV RdRP could interact with an essential autophagy-related protein, SlBeclin1. RdRP, SlHAKAI, and SlBeclin1 interaction complexes form bright granules in the cytoplasm. Silencing of *Beclin1* in *Nicotiana benthamiana* plants abolishes the RdRP-mediated degradation of SlHAKAI, indicating the requirement of Beclin1 in this process. This study uncovers that the PepMV RdRP exploits the autophagy pathway by interacting with SlBeclin1 to promote the autophagic degradation of the SlHAKAI protein, thereby inhibiting the m^6^A modification-mediated plant defense responses.

**Supplementary Information:**

The online version contains supplementary material available at 10.1007/s42994-023-00097-6.

## Introduction

Pepino mosaic virus (PepMV), a member of the genus *Potexvirus*, was first reported in pepino (*Solanum muricatum*) in Peru (Jones et al. 1980) and later in tomato crops around the globe (Hanssen and Thomma 2010; Amer and Mahmoud 2020; He et al. 2020). PepMV can infect many *Solanaceae* species in nature, which causes mild and severe mosaics, bubbling, laminal distortions, and stunting (Hanssen et al. 2008, 2009; Pagan et al. 2006) in leaves or fruit marbling, discoloration, “open fruit” in fruits (Hanssen et al. 2008; Spence et al. 2006). The PepMV genome consists of a single, positive-sense, 6400-nucleotide (nt) RNA strand, encoding a 164-kDa RNA-dependent RNA polymerase (RdRP), three triple gene block (TGB) proteins of 26-, 14-, and 9-kDa (assigned TGBp1, TGBp2, and TGBp3, respectively) and a 25-kDa coat protein (CP) (Gómez et al. 2009). PepMV is highly contagious in tomato plants, as it easily spreads by the standard crop handling procedures through contaminated tools, hands, and clothing and by direct plant-to-plant contact, making it a challenge to tomato production (Spence et al. 2006).

HAKAI, also known as Casitas B-lineage lymphoma-transforming sequence-like protein 1 (CBLL1), was initially identified as a RING finger-type E3 ubiquitin-ligase for the E-cadherin complex in mammals, which bound to the cytoplasmic domain of E-cadherin and mediated its ubiquitination and degradation (Fujita et al. 2002; Pece and Gutkind 2002). Recently, HAKAI as an m^6^A writer has been confirmed in *Arabidopsis* and *Drosophila* (Růžička et al. 2017; Wang et al. 2021; Bawankar et al. 2021). m^6^A, catalyzed by methyltransferases (writers), removed by demethylases (erasers), and recognized by m^6^A binding proteins (readers), is the most pivotal internal modification and is widely present in rRNAs, mRNA, tRNAs, miRNA, and long non-coding RNA (Yue et al. 2019). Increasing evidence shows that m^6^A modification also regulates viral life cycles, and its roles in the arms race of hosts and viruses have been revealed in many reports (Courtney et al. 2017; Ye et al. 2017; Imam et al. 2018; Hao et al. 2019). In the interactions between plants and viruses, AtALKBH9B was first identified as an m^6^A demethylase in *Arabidopsis*, which interacted with the coat protein (CP) of Alfalfa mosaic virus (AMV). The m^6^A methylation of viral RNA limits the infectivity of AMV to the point, where systemic spread into some tissues is nearly entirely blocked if the virus cannot use the host-encoded m^6^A demethylase to achieve demethylation (Martínez-Péreza et al. 2017). Tobacco mosaic virus (TMV) infection reduced the m^6^A level in *Nicotiana tabacum* and promoted ALKBH5-dependent m^6^A demethylation using a UHPLC-HR-MS/MS method (Li et al. 2018b). A recent report revealed the dynamics of m^6^A modification during the interaction between rice and rice stripe virus (RSV) or rice black-streaked dwarf virus (RBSDV), which may act as a primary regulatory strategy in gene expression (Zhang et al. 2021). However, whether and how m^6^A modification regulates virus infection in tomato plants has not been investigated.

Autophagy is a highly conserved intracellular degradation system in eukaryotes for the degradation and recycling of cytoplasmic damaged proteins and cellular organelles (Klionsky and Codogno 2013; Lamb et al. 2013). So far, a series of autophagy-related genes (*ATGs*) have been identified in plants (Thompson and Vierstra 2005; Yoshimoto et al. 2010; Zhou et al. 2015). ATGs function individually or synergistically during the initiation, elongation, and closure of autophagosome formation (Ding et al. 2018; Soto-Burgos et al. 2018). In plants, autophagy was consolidated as a critical component of plant antiviral immunity, which targets viral proteins to autophagosomes for degradation (Nakahara et al. 2012; Hafren et al. 2017, 2018; Haxim et al. 2017; Li et al. 2018a). However, recent reports have revealed that plant viruses could also manipulate or inhibit autophagy via different strategies to counter plant defense (Li et al. 2017, 2020; Yang et al. 2018, 2022; Zhou et al. 2021). Whether plant viruses could manipulate autophagy to counteract m^6^A modification-mediated defense responses in the arms race of host-virus is largely obscure.

In this study, we demonstrate that SlHAKAI from *Solanum lycopersicum*, an m^6^A writer, is involved in anti-PepMV defense in tomato plants. The RdRP encoded by PepMV could interact with SlHAKAI and promote its protein degradation. Furthermore, we show that the PepMV RdRP exploits the autophagy pathway by directly interacting with SlBeclin1 to mediate the autophagic degradation of SlHAKAI. These results suggest that a viral protein could exploit the autophagy factor to compromise the m^6^A-mediated antiviral response, which is a novel virulence strategy during the arms race between plants and viruses.

## Materials and methods

### Plant materials and growth conditions

Seeds of tomato (*Solanum lycopersicum*) and *Nicotiana benthamiana* from our lab were cultivated in soil and incubated in an insect-free growth chamber at 25 °C and 40% relative humidity under a 16 h/8 h light/dark photoperiod. The transgenic RFP-H2B line was a gift from Michael M. Goodin (University of Kentucky, USA). The tomato variety used in this study was Micro-Tom (MT), and the SlHAKAI overexpression and *SlHAKAI* CRISPR/Cas9 mutant plants were generated in an MT background.

### Plasmid construction and plant transformation

The Gateway system (Invitrogen) and the enzyme digestion connection method were used to construct recombinant plasmids. The full-length coding sequences of SlHAKAI and its truncated mutants SlHAKAI-N, SlHAKAI-M, and SlHAKAI-C were cloned by RT-PCR from the cDNA derived from *Solanum lycopersicum* leaves, while PCR cloned the full-length RdRP and its domains Met, Hel, and RdRP2 of PepMV (KY031324) from PepMV infectious clone previously constructed using the specific primer pairs. (Supplementary Table 1). dGDD, the mutant of RdRP2 lacking the GDD motif, was cloned by overlapping PCR with primers listed in Supplementary Table 1. Briefly, the genes mentioned above were amplified by PCR using TransStart^®^ FastPfu DNA Polymerase (TransGen Biotech, China). The resulting DNA fragments were purified (E.Z.N.A. Gel Extraction Kit) and transferred into the entry vector pDONR221 (Invitrogen) by recombination using BP Clonase^®^ (Invitrogen). Insertions in the resulting pDONR clones were verified by sequencing. Then, the linearized fragments using the restriction endonuclease *Mlu*I were transferred into modified Gateway-compatible and gateway-compatible vectors to generate corresponding expression vectors. These plasmids, including pGADT7-DEST (AD), pGBKT7-DEST (BD), pEarleygate201-YN, pEarleygate202-YC, pEarleyGate-101 (101, C-terminal YFP), pEarleyGate102 (CFP in the C terminal), pEarleyGate104 (YFP in the N terminal) and pBA-Flag-Myc4 (Myc tag in the N terminal), were constructed in this study. TRV-based recombinant VIGS vectors (pTRV2-NbATG7, pTRV2-NbBeclin1) were described in our previous report (Li et al. 2018a). To obtain the SlHAKAI overexpression vectors, the full-length of *SlHAKAI* purified above was digested with restriction endonucleases *Kpn* I/*Bam*H I and ligated to the linearized pCambia1300-GFP (C-terminal GFP) with T4 DNA ligase (TransGen Biotech, China). The SlHAKAI-Cas9-sgRNA construct was obtained as followings: the ideal spacer was referred to the CRISPR-P online website (http://cbi.hzau.edu.cn/cgi-bin/CRISPR). SgRNA sequence is CTTCCGGTAGCCAAGAGCCTTGG. SlHAKAI-spacer1-F/R primers were designed according to the sequence of sgRNAs (Supplementary Table 1). After annealing, the double fragments were directly connected with the BKG01 vector (linearized in advance by *Eco31* I) to obtain BKG01-SlHAKAI. After DNA sequencing was confirmed, *Agrobacterium*-mediated tomato transformation was carried out at BIOGLE Gene Tech Co., Ltd. (Jiangsu, China).

### Agroinfiltration and viral inoculation

For viral infection analysis, 3-week-old tomato plants were used for virus inoculation. The infectious clones of PepMV were transformed into *A. tumefaciens* (strain GV3101) by electroporation. The transformed *Agrobacterium* cultures were grown individually until approximately OD_600_ =  ~ 2.0. Then, the cultures were collected and re-suspended to OD_600_ = 1.0 using infiltration buffer (10 mM MgCl_2_, 100 mM MES (pH 5.7), 2 mM acetosyringone) for 3 h at room temperature. The suspensions were infiltrated into cotyledons of tomatoes using 1-mL syringes. Inoculated plants were photographed with a Canon 400D digital camera at different periods. The PepMV infection assay was repeated at least three times. For transient expression analysis in *N. benthamiana* leaves, constructs generated were transformed into *A. tumefaciens* strain EHA105 via electroporation. The wild-type or RFP-H2B transgene *N. benthamiana* plants in the 4–5 leaf stage were used for *Agrobacterium*-mediated transient expression with minor modifications of our previous procedures (Li et al. 2018a). *Agrobacterium* cultures harboring pTRV1 and pTRV2-VIGS (TRV2-GUS, TRV2-NbATG7, TRV2-NbBeclin1) were resuspended for the TRV-VIGS assay in infiltration buffer and mixed at a 1:1 ratio. The mixed *A. tumefaciens* cultures were infiltrated into leaves of *N. benthamiana* plants at the 4–5 leaf stage after 3 h incubation at room temperature. As an indicator, *N. benthamiana* plants infiltrated with TRV-based VIGS vectors targeting phytoene desaturase (PDS). TRV-GUS- or TRV-VIGS-treated plants were agroinfiltrated with PepMV infectious clones when the silenced phenotype of TRV-PDS-treated plants appeared.

### Y2H, BiFC, subcellular localization

Y2H was performed according to the Clontech yeast protocol. Briefly, plasmids expressing viral and host proteins were co-transformed into yeast cells (strain Y2H Gold, Clontech catalog number: 630498). Then, selective culture mediums lacking tryptophan and leucine (SD/-Trp-Leu) or tryptophan, leucine, histidine, and adenine (SD/-Trp-Leu-His-Ade) were plated with yeast cells mentioned above to confirm the right transformation or analyze the interactions. BiFC and subcellular localization experiments were performed as previously described (Li et al. 2018a). After 36–72 h infiltration, 1–2 cm^2^ leaf sections were excised for examining fluorescence in epidermal cells by confocal microscopy (Carl Zeiss 980, German), equipped with a 63 × water-corrected objective in multitrack mode. CFP was excited at 458 nm and captured at 470–500 nm, YFP was excited at 514 nm and captured at 565–585 nm, and RFP was excited at 543 nm and captured at 590–630 nm. In general, at least 20 cells were examined for each experiment. The sequential scanning mode was applied for the co-imaging of different fluorescent proteins. Images were collected and analyzed using ZEN 2 (Carl Zeiss Microscope GmbH2011) imaging software.

### RNA extraction and qRT-PCR analysis

The total RNAs of plant leaves were extracted using TRIzol reagent (Invitrogen, USA, Cat. no. 15596-026) according to the manufacturer’s protocols. The extracted RNAs were then reverse-transcribed with PrimeScript™ 1st Strand cDNA Synthesis Kit (TaKaRa) using oligo (dT) primers after removing gDNA. cDNA synthesized from reverse transcription of RNA samples was used to determine the mRNA expression levels of target genes and to quantify PepMV accumulation levels at the date indicated. *NbActin* or *SlActin* was used as an internal control for *N. benthamiana* or tomato. Primers used in this study are provided in Supplementary Table 1.

### Western blotting analysis

The plant tissues to be tested were extracted with SDS lysis buffer (100 mM Tris–HCl, pH = 6.8, 10% SDS) for Western blotting analysis as previously described (Li et al. 2018a). Western blotting was performed with primary mouse polyclonal antibodies, followed by goat anti-mouse secondary antibody conjugated to horseradish peroxidase (Bioeasytech, no. YM6704). Anti-PepMV CP polyclonal antibody at 1:10,000 dilution was used for diagnosis of PepMV-infection. Anti-GFP antibody (1:5000; Abcam, no. ab6556) and anti-Myc antibody (1:5000; Abcam, no. ab32) were used to diagnose the target protein. Blotted membranes were washed 3 times (5 min each time) thoroughly with PBST buffer (137 mM NaCl, 2.7 mM KCl, 4.3 mM Na_2_HPO_4_·7H_2_O, 1.4 mM KH_2_PO_4_, 0.05% Tween 20) and visualized using chemiluminescence according to the manufacturer’s protocol (ECL; GE Healthcare). Total proteins were stained with Coomassie Brilliant Blue R-250 (CBB) to show equal loading. The experiments in this study were repeated at least three times with similar results.

### Chemical treatments

Phosphate-buffered saline buffer (137 mM NaCl, 2.7 mM KCl, 4.3 mM Na_2_HPO_4_·7H_2_O, 1.4 mM KH_2_PO_4_) containing 2% dimethyl dulfoxide (DMSO; control) or 2% 5 mM 3-MA (Sigma) for inhibition of autophagy, or 2% (100 µM) MG132 (Sigma-Aldrich) for inhibition of the 26S proteasome, was infiltrated into leaves 8–12 h before leaves were collected.

### Extraction of PepMV particles

Nearly 20 g of tomato leaves infected with PepMV were used for viral particle extraction at 15 dpi. The fresh leaves and 40 mL lysis buffer (0.5 mol/L Sodium Phosphate Buffer (pH 7.5), 0.01 mol/L Na-EDTA, 0.1% β-mercaptoethanol) were mixed and grounded with a homogenizing machine for 5 min. The mixture was further filtered with double-layer gauze and centrifuged for 20 min at 6000 r/min. 7% PEG, 2.5% Triton X-100 and 0.1 mol/L NaCl were subsequently added to the supernatant obtained in the last step and stirred at 4 °C for 4 h. Then, the mixture was centrifuged for 15 min at 11,000 r/min, and the precipitation was retained and washed 3 times with 0.02 mol/L Sodium phosphate buffer (PH 7.5). The supernatant obtained in the last step was then added to the 35-mL unique thin-wall centrifugal tube for ultra-speed centrifuge with a 5 mL 30% sucrose pad at the bottom of the tube and subjected to 35,000 r/min centrifugation for 100 min. The precipitation was resuspended with 0.02 mol/L sodium phosphate buffer (PH 7.5), and the resuspension was just the crude extract of PepMV particles.

### m^6^A dot blot assay

First, RNA was made a serial dilution to 400 ng/μL, 200 ng/μL, and 50 ng/μL using RNase-free water (PepMV particles to 1 µg/μL, 0.5 µg/μL, 0.2 µg/μL with 0.02 mol/L sodium phosphate buffer) and denatured at 90 °C for 3 min. Chilling on ice immediately after denaturation, 2 μL mRNA or PepMV particles were dropped on the Hybond-N + membrane optimized for nucleic acid transfer. Then, the membrane was cross-linked in a Stratalinker 2400 UV Crosslinker by UV light twice using the Autocrosslink mode for 1 min, washed with 10 mL wash buffer (1 × PBS, 0.02% Tween-20), and incubated in 10 ml of blocking buffer (1 × PBS, 0.02% Tween-20, 5% non-fat milk) for 1 h at room temperature with gentle shaking. Afterward, the membrane was incubated with the anti-m^6^A antibodies (1:250 dilution; 2 μg/mL) in 10 mL of antibody dilution buffer (1 × PBS, 0.02% Tween-20, 5% non-fat milk) overnight at 4 °C with gentle shaking. At last, the imaging system visualized the membrane after incubation with the secondary antibodies for 1 h at room temperature. RNA was stained with 0.1% Methylene blue (MB; Solarbio, China, Cat#G1300), while PepMV particles were blotted with PepMV CP antibodies to show equal loading. The experiments in this study were repeated at least three times with similar results.

## Results

### SlHAKAI is involved in antiviral defense in *Solanum lycopersicum*

**Fig. 1:**
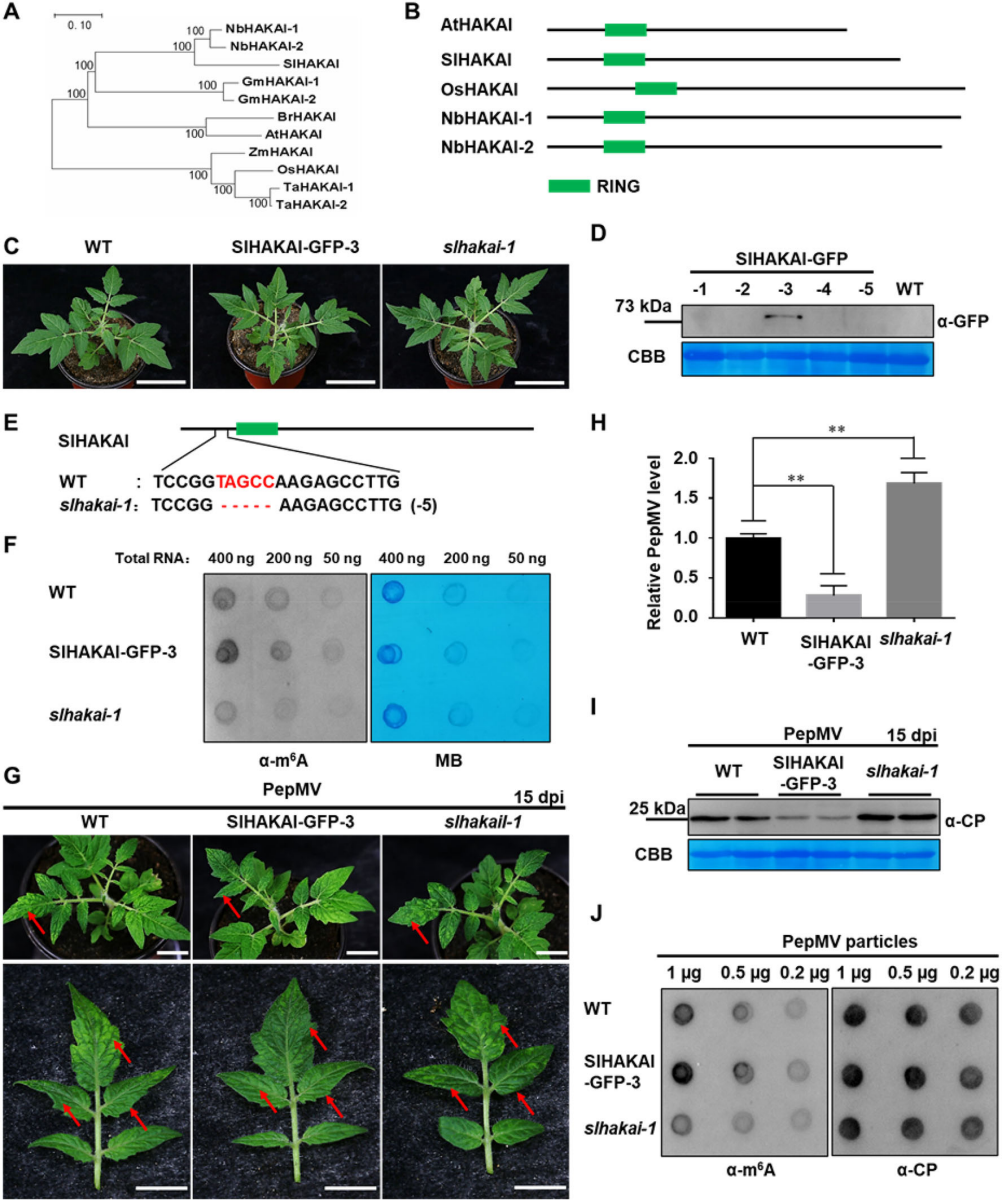
SlHAKAI negatively regulates tomato resistance to PepMV. **A** Phylogenetic analysis is shown for amino acid sequences of HAKAI proteins from 8 representative plant species. Sequence alignments and tree construction were conducted in MEGA7 using the Neighbour-Joining method. NbHAKAI-1, 2, HAKAIs from *Nicotiana benthamiana*; SlHAKAI, HAKAI from *Solanum lycopersicum*; GmHAKAI-1, 2, HAKAIs from *Glycine max*; BrHAKAI, HAKAI from *Brassica rapa*; AtHAKAI, HAKAI from *Arabidopsis thaliana*; ZmHAKAI, HAKAI from *Zea mays* L.; OsHAKAI, HAKAI from *Oryza sativa* L.; TaHAKAI-1, 2, HAKAIs from *Triticum aestivum* L. **B** Domain compositions of HAKAI proteins in AtHAKAI, SlHAKAI, OsHAKAI, and NbHAKAI. All HAKAI proteins contain a conserved RING domain. **C** The phenotypes of SlHAKAI-overexpression (SlHAKAI-GFP-3) transgene and *SlHAKAI*-knockout (*slhakai-1*) plants in the T2 generation. WT: wild-type (WT). The indicated plants were photographed 30 days after seeding. White bar represents 5 cm. **D** Western blot analysis of the SlHAKAI-GFP protein accumulation in SlHAKAI-GFP-overexpression transgenic tomato plants using anti-GFP antibodies. SlHAKAI-GFP-1, 2, 3, 4, 5 represents the 5 individual transgene lines. Coomassie Brilliant Blue R-250 (CBB)-stained Rubisco large subunit was set as a loading control. **E** DNA sequencing and sequence alignment were conducted to confirm the mutation in the *SlHAKAI*-knockout (*slhakai-1*) transgene plants. This nucleotide deletion caused a frameshift mutation of *SlHAKAI.* **F** Dot blotting analysis of the overall levels of m^6^A modification in WT, SlHAKAI-GFP-3, and *slhakai-1* tomato plants using anti-m^6^A antibodies. Methylene blue (MB) staining of total RNA served as an equal loading. **G** The pepino mosaic virus (PepMV) caused symptoms in WT, SlHAKAI-GFP-3, and *slhakai-1* tomato plants. The PepMV-inoculated tomato plants were photographed at 15 dpi. The white bar represents 2 cm. **H** Relative viral accumulations of PepMV in the plants shown in (G) were detected by qRT-PCR at 15 dpi. *SlActin* was used as the internal reference gene to normalize the relative expression, and the value in WT tomato plants was set to 1. Values represent the mean ± SD from 3 independent biological samples. Student’s *t*-test was used to analyze each data group, and double asterisks indicate significant statistical differences (**P < 0.01) between the two treatments. **I** The accumulation levels of PepMV protein in (G)-indicated plants were determined by Western blotting using anti-PepMV CP antibodies at 15 dpi. CBB-stained Rubisco large subunit was a loading control. **J** Dot blotting was used to detect m^6^A modification levels with PepMV particles extracted from PepMV-infected WT, SlHAKAI-GFP-3, and *slhakai-1* plants indicated in (G) using anti-m^6^A antibodies. Blotting with antibodies of PepMV CP indicated the loading amount of PepMV particles

Although MTA, MTB, VIRILIZER, and FIP37 are essential members of the m^6^A writer complex, knockout of *MTA*, *MTB*, *VIRILIZER*, or *FIP37* led to embryo lethal in *Arabidopsis* (Růžička et al. 2017). In this study, we either failed to get the *MTA*-, *MTB*-, *VIRILIZER-,* or *FIP37*-knockout tomato plants. Thus, we chose HAKAI as the research object. HAKAI is a conserved component of the methyltransferase complex in eukaryotes (Bawankar et al. 2021). In *Arabidopsis*, HAKAI is also functionally required for complete m^6^A mRNA methylation (Růžička et al. 2017). By constructing evolutionary trees and analyzing functional domains of the HAKAI proteins in different plant species, we found that the HAKAIs from several plant species share a high identity in amino acid sequences. All of them have a conserved RING domain (Fig. 1A, B). To investigate whether HAKAI influences PepMV infection in tomato plants, SlHAKAI-GFP transgenic plants were generated and obtained via the *Agrobacterium*-mediated leaf disc method (Sun et al. 2006). Line 3 (SlHAKAI-GFP-3) and line 8 (SlHAKAI-GFP-8) of the SlHAKAI-overexpression tomato plants showed high levels of SlHAKAI-GFP by Western blot analysis using anti-GFP antibodies (Fig. 1D and Fig. S1B). At the same time, we used CRISPR/Cas9 technology to obtain *SlHAKAI*-knockout tomato lines (*slhakai-1* and *slhakai-4*). *slhakai-1* carried five nucleotide deletions (Fig. 1E), while *slhakai-4* carried one nucleotide insertion near the cleavage site (Fig. S1C). SlHAKAI-overexpression and *SlHAKAI*-knockout tomato plants displayed similar growth, development, and fruit phenotypes with wild-type (WT) tomato plants (Fig. 1C, Fig. S1A, and data not shown), which was consistent with the fact that both two *hakai* alleles resemble WT in *Arabidopsis* plants (Růžička et al. 2017). In T2 generation tomato plants, overexpression of SlHAKAI and knockout of *SlHAKAI* increased and decreased the total m^6^A levels compared to WT using m^6^A dot blotting analysis, respectively (Fig. 1F). This result confirmed that SlHAKAI played a role in m^6^A modification in tomato plants. Then, the SlHAKAI-GFP transgenic and *slhakai* tomato plants in the T2 generation were inoculated with PepMV infectious clones by agroinfiltration. The inoculated plants were maintained to monitor symptom development. After inoculating these plants with PepMV at 15 days, WT tomato plants showed typical and obvious mosaic symptoms (Fig. 1G and Fig. S1D). SlHAKAI-overexpression plants displayed delayed symptom development and milder mosaic phenotypes than WT plants (Fig. 1G and Fig. S1D). In contrast, significant severe mosaic leaf symptoms appeared on the *SlHAKAI-*knockout tomato plants (Fig. 1G and Fig. S1D). The newly emerged leaves with mosaic symptoms were then extracted for RNA and protein. As shown in Fig. 1H, I, fewer viral RNA and coat protein (CP) accumulations of PepMV were found in SlHAKAI-overexpression transgenic tomato plants compared to the WT plants. Consistently, more viral RNA and CP accumulated in the *SlHAKAI*-knockout tomato plants compared to the WT plants (Fig. 1H, I and Fig. S1E). Furthermore, the viral particles from PepMV-infected WT, SlHAKAI-overexpression, and *SlHAKAI*-knockout tomato plants were extracted and purified for viral genome m^6^A detection at 15 dpi. Dot blot analysis using anti-m^6^A antibodies revealed that the viral RNA from SlHAKAI-overexpression and *SlHAKAI*-knockout tomato plants exhibited more and fewer m^6^A levels than that from WT plants, respectively (Fig. 1J). These findings indicate that SlHAKAI negatively regulates PepMV infection, probably through m^6^A modifications of viral RNA.

### PepMV RdRP interacts with SlHAKAI

**Fig. 2:**
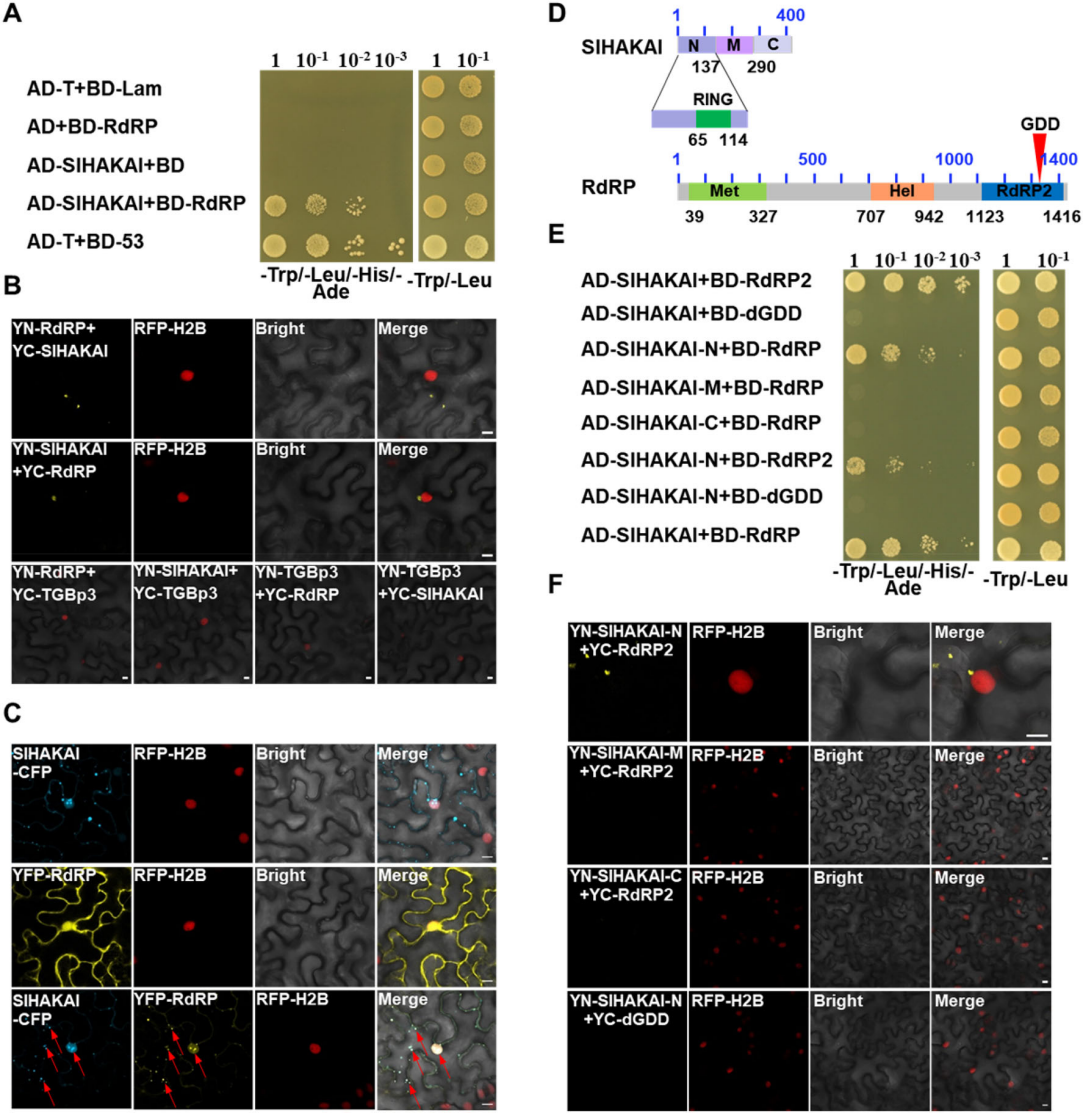
SlHAKAI interacts with PepMV RdRP. **A** Y2H assays of the interaction between SlHAKAI and RdRP. Yeast cells co-transformed with AD-T7-T + BD-T7-53 serve as a positive control; yeast cells co-transformed with AD-SlHAKAI and the empty BD, with the empty AD and BD-RdRP, or with AD-T + BD-Lam are negative controls. **B** Confirmation of the SlHAKAI-RdRP interaction by BiFC assay. BiFC assays between SlHAKAI and RdRP in the leaves of RFP-H2B (red) transgenic *N. benthamiana*. Confocal imaging was performed at 48 hpi. SlHAKAI and RdRP were fused to the N (YN) and C-terminal (YC) fragments of yellow fluorescent protein (YFP). The yellow fluorescence indicated the SlHAKAI-RdRP. Bars, 10 μm. **C** Co-localization of SlHAKAI-CFP and YFP-RdRP in the leaf cells of RFP-H2B transgenic *N. benthamiana* by confocal microscopy at 48 hpi. Arrows indicate the overlapping fluorescence of SlHAKAI-CFP and YFP-RdRP. Bars, 10 μm. **D** Schematic representation of full-length and truncated proteins of SlHAKAI and RdRP. The conserved domains in SlHAKAI and RdRP are indicated. RING: RING Ubox domain; Met: methyltransferase domain; Hel: helicase domain; RdRP2: RdRP2 domain. **E** Mapping the interaction domain between SlHAKAI and RdRP by Y2H assays. Y2H Gold yeast cells co-transformed with the indicated plasmids were subjected to tenfold serial dilutions and plated on synthetic dextrose (SD)/-Trp, -Leu, -His, -Ade or SD/-Trp, -Leu medium to screen for possible interactions at 3 days after transformation (A and E). **F** BiFC assays of the domains of SlHAKAI and RdRP. Confocal images were taken at 48 h post infiltration (hpi). The nuclei of *N. benthamiana* leaf epidermal cells are marked by RFP-H2B (red). These experiments were repeated three times independently. At least 20 cells per sample were observed, and representative results were displayed. Bars, 10 μm (**B**, **C**, and **F**)

SlHAKAI played an anti-viral role during PepMV infection, so we wondered whether viral proteins could inhibit or manipulate SlHAKAI to counter-defend SlHAKAI-mediated anti-viral responses. Thus, possible interactions between SlHAKAI and the 5 PepMV-encoded viral proteins were screened by yeast two-hybrid (Y2H) assays. Positive protein–protein interaction was found between SlHAKAI and PepMV RdRP, and no interaction was observed between SlHAKAI with any other PepMV proteins (Fig. 2A and Fig. S2A). The RdRP-SlHAKAI interaction was further examined by bimolecular fluorescence complementation (BiFC) in RFP-H2B (a nuclear marker) transgenic *Nicotiana benthamiana* leaves. The BiFC assays of the interaction between YN-RdRP and YC-SlHAKAI, or YN-SlHAKAI and YC-RdRP was observed by confocal microscopy at 48 hpi, which formed bright granules in the cytoplasm (Fig. 2B, lines 1, 2). No interaction was observed between SlHAKAI and TGBp3 (PepMV TGBp3, as a negative control) (Fig. 2B, line 3). The BiFC assays of the interaction between YN-SlHAKAI and YC-RdRP upon PepMV infection were also conducted. We found that PepMV infection did not affect the formation of the SlHAKAI-RdRP interaction granules in the cytoplasm (Fig. S3A). SlHAKAI-CFP formed specific punctate-structure dots in the cytoplasm and nucleus, whereas RdRP was uniformly distributed in the cytoplasm and nucleus (Fig. 2C, lines 1, 2). When SlHAKAI-CFP and YFP-RdRP were co-expressed, the localization of YFP-RdRP was redistributed to the SlHAKAI-CFP-labeled punctuate structures in the cytoplasm and nucleus at 48 hpi (Fig. 2C, line 3). In addition, PepMV infection did not influence the localization of SlHAKAI and RdRP either (Fig. S2C). These results suggested that PepMV RdRP could interact with SlHAKAI to form an aggregated interaction complex in the cytoplasm and nucleus in planta.

We next mapped the protein domains required for the interaction between SlHAKAI and RdRP. SlHAKAI contains a conserved N-terminal RING domain, while RdRP contains three conserved domains, including Met (the viral methyltransferase domain), Hel (the viral RNA helicase domain), and RdRP2 (the viral RdRP2 domain) (Fig. 2D). Then, SlHAKAI and RdRP were divided into three fragments based on their domain compositions (SlHAKAI- N, M, C, and RdRP- Met, Hel, RdRP2). SlHAKAI-N includes a RING domain, and no specific motif exists in SlHAKAI-M or SlHAKAI-C domain (Fig. 2D). Y2H revealed that the N-terminal RING domain of SlHAKAI interacted with the RdRP2 domain of RdRP (Fig. 2E and Fig. S3A). SlHAKAI-N and SlHAKAI displayed similar subcellular localization patterns forming bright cytoplasm spots (Fig. S3B). RdRP and RdRP2 fused YFP both localized in the cytoplasm and nucleus (Fig. S3C). Consistently, PepMV infection did not influence the sublocalization patterns of the domains of SlHAKAI and RdRP (Fig. S3B, C). Since the highly conserved GDD motif required for the RdRP activity is located in the domain of RdRP2 of RdRP (Li et al. 2018a), we tested the importance of this motif to the SlHAKAI-RdRP interaction. As expected, the deletion of GDD (dGDD) abolished the interaction between SlHAKAI and RdRP (Fig. 2E). This observation was consistent with results from BiFC assays *in planta* (Fig. 2F, line 4).

### PepMV RdRP promotes the SlHAKAI degradation via autophagy

**Fig. 3:**
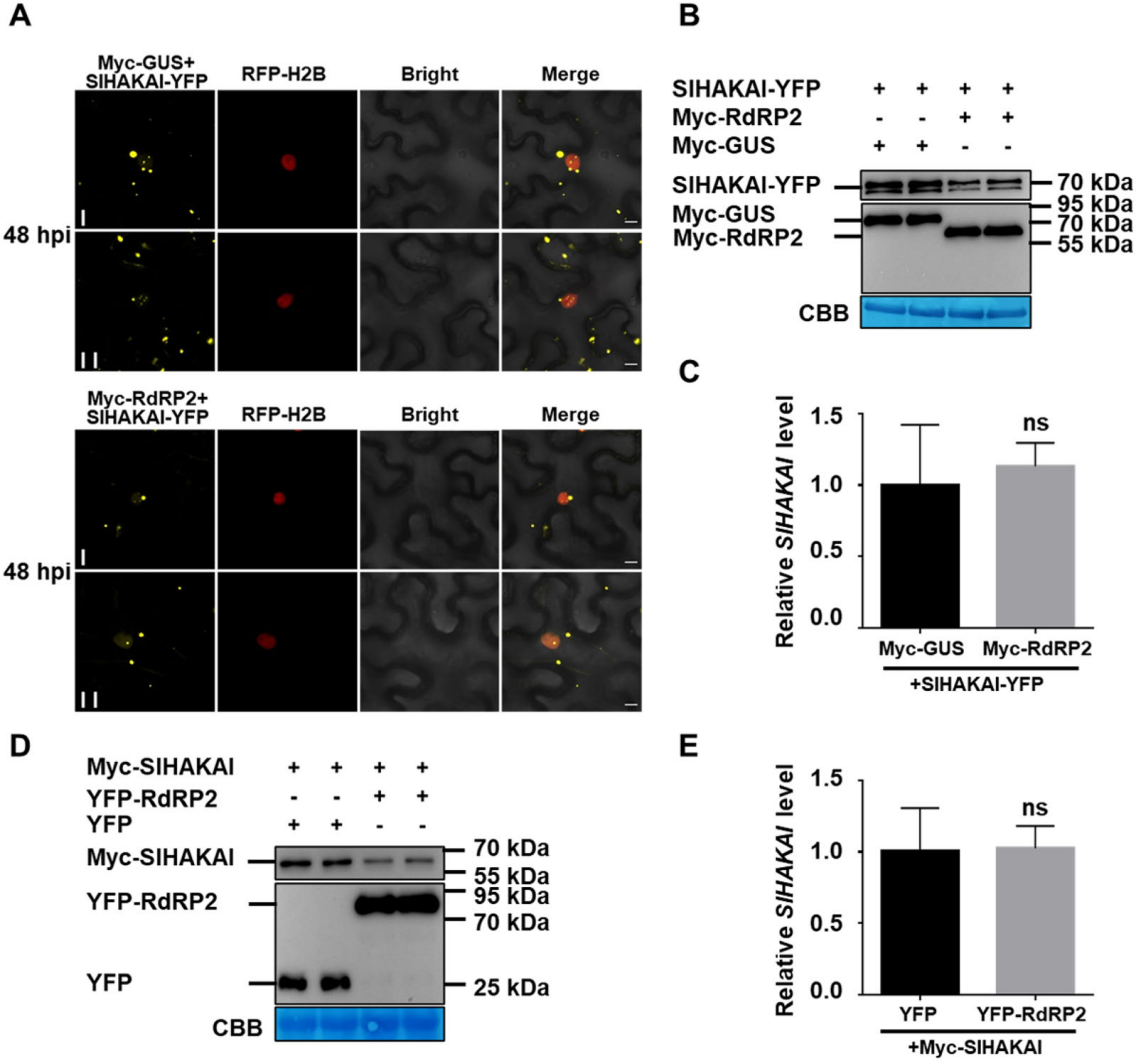
RdRP promotes the degradation of SlHAKAI. **A** Confocal micrographs showing RFP-H2B transgene *N. benthamiana* leaf cells co-infiltrated with *Agrobacterium* carrying SlHAKAI-YFP and Myc-GUS or Myc-RdRP2 at 48 hpi. Bars, 10 μm. **B** Immunoblotting analysis of total protein extracted from leaves indicated in (A) at 48 hpi with anti-GFP or anti-Myc antibodies. **C** Relative expression levels of *SlHAKAI* in (A)-indicated leaves at 48 hpi. **D** Immunoblotting analysis of total protein isolated from leaves co-infiltrated with *Agrobacterium* carrying Myc-SlHAKAI and YFP or YFP-RdRP2 at 48 hpi with anti-Myc or anti-GFP antibodies. CBB-staining of Rubisco large subunit was set as a loading control (**B** and **D**). **E** Relative expression levels of *SlHAKAI* in (D)-indicated leaves at 48 hpi. *NbActin* was used as the internal reference gene to normalize the relative expression, and the value in Myc-GUS and YFP-RdRP2 (**C**) or YFP and Myc-SlHAKAI (**E**) co-expressed *N. benthamiana* leaves was set to 1. Values represent the mean ± SD from 3 independent biological samples. Student’s *t*-test was used to analyze each data group, and ns represents no significance between the two treatments (**C**, **E**)

To investigate the interaction function between SlHAKAI and RdRP, SlHAKAI-YFP was co-expressed with Myc-GUS (mock) or Myc-RdRP2 in RFP-H2B transgenic *N. benthamiana* plants. At 48 hpi, the intensity of fluorescence and the size and quantity of granules of SlHAKAI were significantly reduced when co-expressed with Myc-RdRP2, compared with the mock (Fig. 3A). Samples above were then collected to extract RNA and protein for qRT-PCR and immunoblotting analysis, respectively. As shown in Fig. 3B, the protein accumulation of SlHAKAI was significantly decreased when co-expressed with YFP-RdRP2. No significant changes in *SlHAKAI* mRNA were observed when expressed with Myc-GUS or Myc-RdRP2 (Fig. 3C). Furthermore, a similar reduction was also observed when YFP-RdRP2 was co-expressed with Myc-SlHAKAI (Fig. 3D, E).

**Fig. 4:**
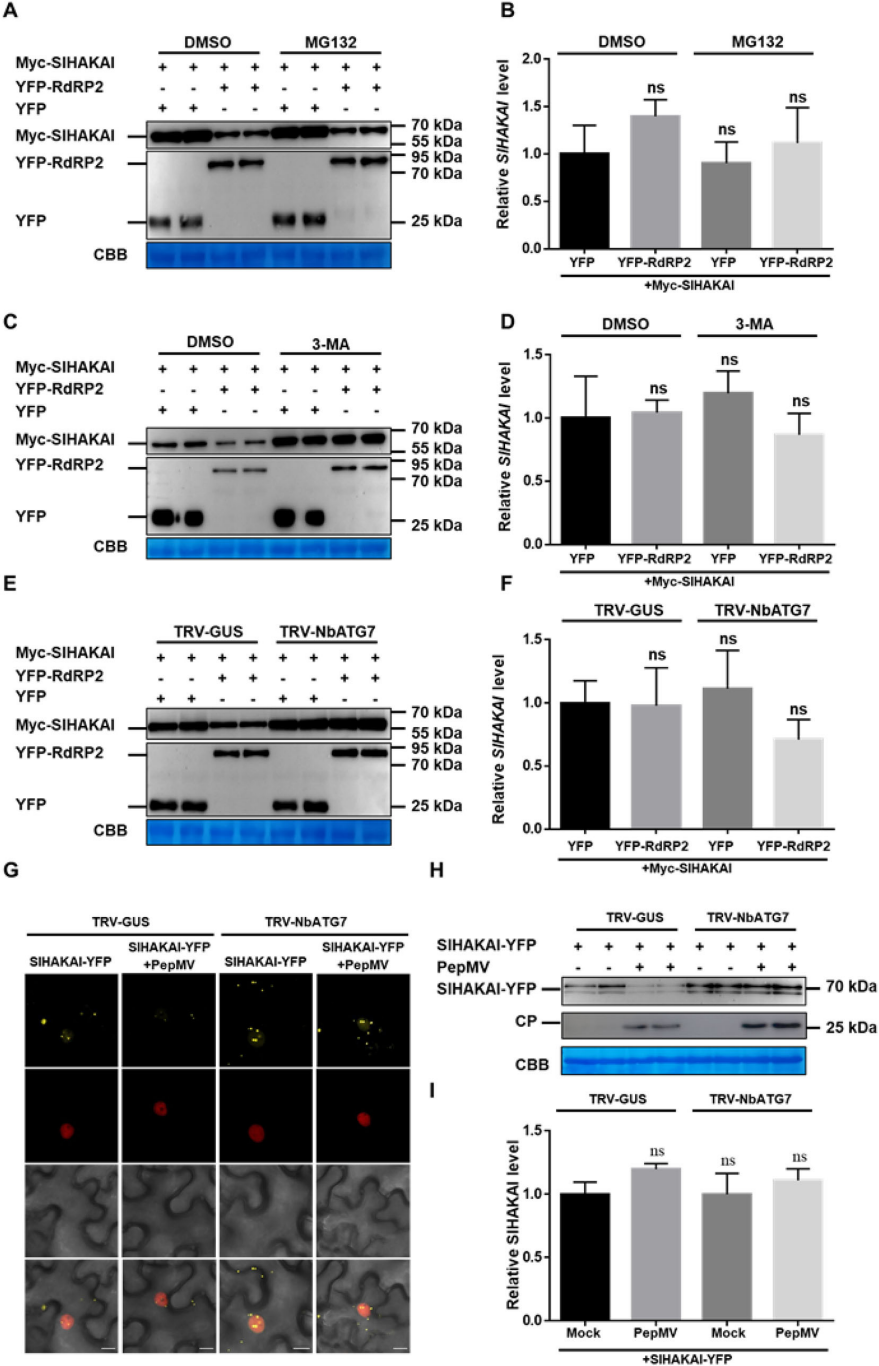
RdRP mediates the autophagic degradation of SlHAKAI. **A** MG132 (an inhibitor of the ubiquitin-26S proteasome system) does not affect the RdRP-mediated degradation of Myc-SlHAKAI. Total protein was isolated from plant leaves co-agroinfiltrated with *Agrobacterium* harboring Myc-SlHAKAI and YFP or YFP-RdRP2, followed by DMSO or MG132 treatment at 48 hpi. **B** Relative expression levels of *SlHAKAI* in (A)-indicated leaves at 48 hpi. **C** Effect of an autophagy inhibitor, 3-MA, on the RdRP-mediated degradation of Myc-SlHAKAI. **D** Relative expression levels of *SlHAKAI* in (C)-indicated leaves at 48 hpi. **E** Effect of silencing of *NbATG7* on the RdRP-mediated degradation of Myc-SlHAKAI. Plants preinoculated with TRV-GUS or TRV-NbATG7 at 10 dpi were co-agroinfiltrated with *Agrobacterium* harboring Myc-SlHAKAI and YFP or YFP-RdRP2 in the upper leaves. Total protein was extracted from infiltrated leaves at 48 hpi. **F** Relative expression levels of *SlHAKAI* in (E)-indicated leaves at 48 hpi (**B**, **D**, **F**). **G** Subcellular localization of SlHAKAI-YFP in the leaf cells of NbATG7-silenced or non-silenced RFP-H2B transgenic *N. benthamiana* plants with or without PepMV infection by confocal microscopy at 48 hpi. Bars, 10 μm. **H** Immunoblotting of SlHAKAI-YFP in (G)-indicated leaves at 48 hpi. Immunoblotting was performed using anti-GFP or anti-Myc antibodies. All immunoblotting assays in this figure were repeated at least three times, and one representative blot was shown. CBB-staining of Rubisco large subunit is a loading control (**A**, **C**, **E**, **H**). **I** Relative expression levels of *SlHAKAI* in (E)-indicated leaves at 48 hpi. *NbActin* was used as an internal control, Student’s *t* test was used to analyze each data group, and ns represents no significance between the two treatments

The most studied proteasome is the primary proteolytic machinery that regulates cellular protein homeostasis (proteostasis) by selectively degrading ubiquitinated proteins (Finley 2009). Given this, drug treatment with MG132, a well-known ubiquitin-26S proteasome system (UPS) inhibitor, was performed to test whether UPS was responsible for the RdRP2-mediated degradation of SlHAKAI. As shown in Fig. 4A, there was no significant difference between DMSO-treated and MG132-treated samples, which indicated that the RdRP2-mediated degradation of SlHAKAI might be UPS-independent. Next, we used an autophagy inhibitor, 3-methyladenine (3-MA), to treat the samples co-expressed with RdRP2 and SlHAKAI. Western blotting analysis revealed that the RdRP2-mediated HAKAI protein degradation was markedly inhibited by treatment with 3-MA (Fig. 4C). No obvious change in *SlHAKAI* mRNA was observed between DMSO- and 3-MA-treated Myc-SlHAKAI and YFP-RdRP2 co-expressing samples (Fig. 4D). Then, a tobacco rattle virus (TRV)-based virus-induced gene silencing (VIGS) system was employed to silence an essential autophagy gene, *NbATG7*, to confirm whether autophagy is involved in the RdRP2-mediated degradation of SlHAKAI (Fig. S4A). As shown in Fig. 4E, F, the knockdown of *NbATG7* remarkably inhibited the degradation of Myc-SlHAKAI. Besides, we also analyzed the expression levels of SlHAKAI upon PepMV infection when *NbATG7* was knocked down. As shown in Fig. 4G, H, PepMV infection reduced the fluorescence intensity and decreased the protein accumulation of SlHAKAI-YFP in TRV-GUS plants. However, the knockdown of *NbATG7* (TRV-NbATG7) remarkably inhibited the degradation of SlHAKAI-YFP upon virus infection. Taken together, PepMV infection promoted the autophagic degradation of SlHAKAI, which RdRP was responsible for this through interacting with SlHAKAI.

### The PepMV RdRP-mediated autophagic degradation of SlHAKAI requires SlBeclin1

**Fig. 5:**
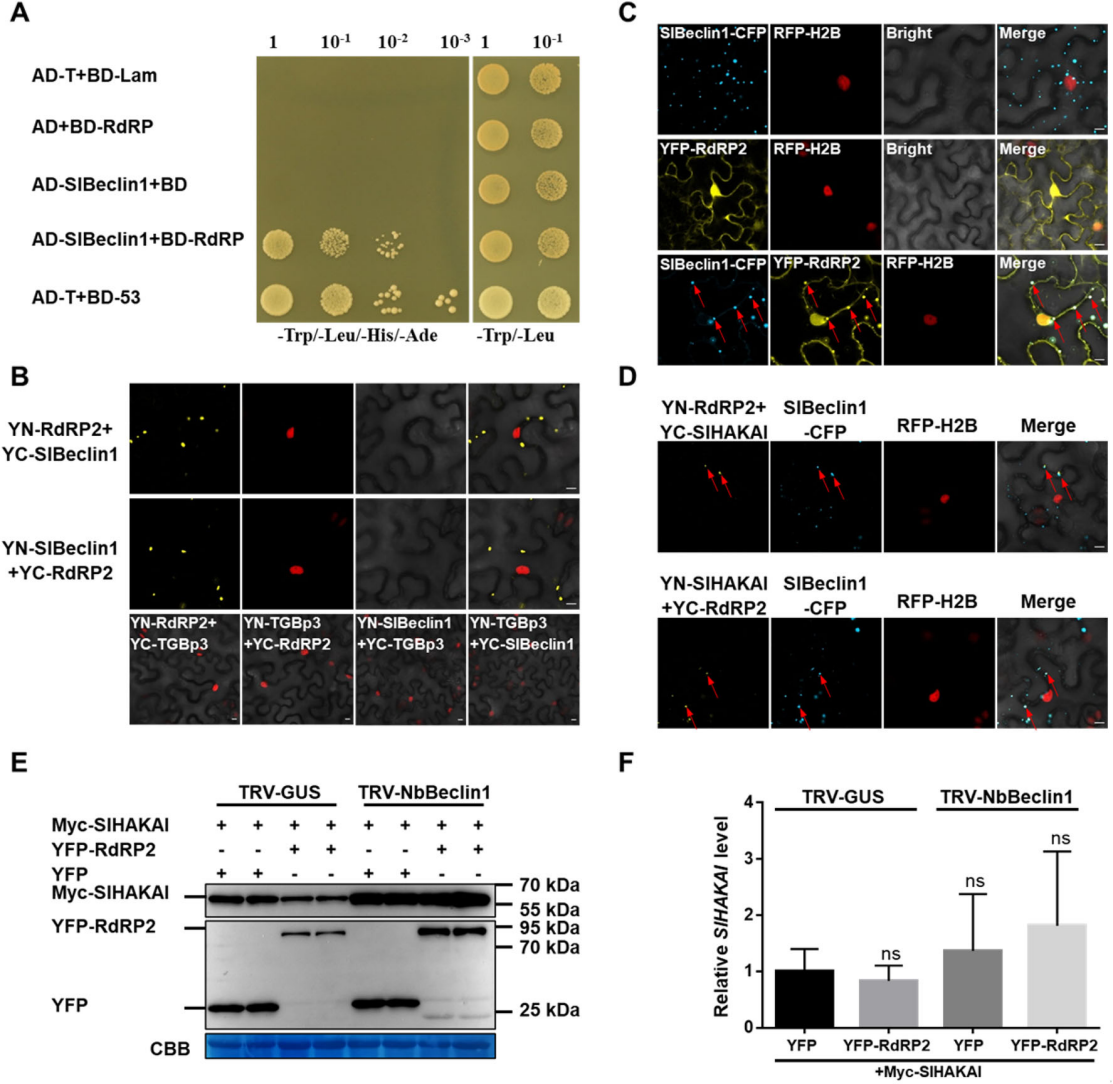
Beclin1 is required for the RdRP-mediated autophagic degradation of SlHAKAI. **A** Y2H assays of the interaction between SlBeclin1 and RdRP. Y2H Gold yeast cells co-transformed with the indicated plasmids were subjected to tenfold serial dilutions and plated on a selective medium to screen for positive interactions 3 days after transformation. The yeast cells co-transformed with AD + BD-RdRP, AD-SlBelin1 + BD, or AD-T + BD-Lam were served as negative controls, and AD-T + BD-53 used as a positive control. **B** Confirmation of SlBeclin1-RdRP interaction by BiFC assay. Bars, 10 µm. **C** By confocal microscopy, the co-localization of SlBeclin1-CFP and YFP-RdRP in the leaf cells of RFP-H2B transgenic *N. benthamiana*. Arrow indicates the overlapping fluorescence, which was produced from SlBeclin1-CFP and YFP-RdRP. Bars, 10 µm. **D** Co-localization of the SlHAKAI-RdRP interaction complex with SlBeclin1. Bars, 10 μm. Confocal images were taken at 48 hpi. The nuclei of *N. benthamiana* leaf epidermal cells are marked by RFP-H2B (red). These experiments were repeated three times independently. At least 20 cells per sample were observed, and representative results were displayed (**B**–**D**). **E** Effect of silencing of *NbBeclin1* on the RdRP-mediated degradation of Myc-SlHAKAI. Plants pre-inoculated with TRV-GUS or TRV-NbBeclin1 at 10 dpi were then co-agroinfiltrated with *Agrobacterium* harboring Myc-SlHAKAI and YFP or YFP-RdRP2. Total protein was extracted from infiltrated leaves at 48 hpi. Immunoblotting was performed using anti-GFP or anti-Myc antibodies. All immunoblotting assays in this figure were repeated at least three times, and one representative blot was shown. CBB staining of Rubisco large subunit serves as a loading control. **F** Relative expression levels of *SlHAKAI* in (E)-indicated leaves at 48 hpi. *NbActin* was used as an internal control, Student’s *t*-test was used to analyze each data group, and ns represents no significance between the two treatments

Our previous report has shown that NbBeclin1 interacted with the RdRPs of several RNA viruses and promoted their degradation (Li et al. 2018a). So, we hypothesized SlBeclin1 might play a role in RdRP-mediated autophagic degradation of SlHAKAI. Y2H assay was conducted to verify whether SlBeclin1 interacted with RdRP. As shown in Fig. 5A, a strong interaction was observed in the selective yeast medium. This interaction was further confirmed by BiFC assay in RFP-H2B transgenic *N. benthamiana* plants, which revealed that this interaction occurs in the cytoplasm (Fig. 5B). As a negative control, no YFP fluorescence was observed when the movement protein TGBp3 from PepMV was co-expressed with SlBeclin1 or RdRP (Fig. 5B). SlBeclin1 was present in the cytoplasm with specific punctate subcellular localization, while RdRP localized to the cytoplasm and nucleus at 48 hpi when expressed alone (Fig. 5C). However, when YFP-RdRP was co-expressed with SlBecline1-CFP, the localization of YFP-RdRP was re-directed to the SlBeclin1-CFP-labeled punctuate structures in the cytoplasm at 48 hpi (Fig. 5C). Of note, the SlHAKAI-RdRP interaction complex was co-localized with SlBeclin1, forming bright granules in the cytoplasm (Fig. 5D). Since Beclin1 is required for autophagy in mammalian and plant cells (Liang et al. 1999; Fujiki et al. 2007), we thus speculate that Beclin1 may direct the SlHAKAI-RdRP complex into autophagosomes for degradation by interacting with RdRP during viral infection. We then silenced *NbBeclin1* using a VIGS vector to determine whether RDRP2-mediated SlHAKAI degradation was affected by the expression of NbBeclin1 (Fig. S4B). As shown in Fig. 5E, F, the RdRP2-mediated degradation of Myc-SlHAKAI was remarkably inhibited in the *NbBeclin1*-silenced cells.

## Discussion

Plants’ successful survival depends on their ability to exploit numerous defense mechanisms against invading pathogens or hostile environments. Here, we demonstrate that SlHAKAI is involved in anti-PepMV defense via m^6^A modification of viral RNA in tomato plants. m^6^A, the most pivotal internal modification in RNAs, is involved in various biological processes in eukaryotes (Yue et al. 2019). Crosstalk between mRNA m^6^A modification and autophagy has been reported in mammals (Jin et al. 2018; Wang et al. 2020; Chen et al. 2021). In autophagy, a critical initial event is the formation of the autophagosome. This unique double-membrane organelle engulfs the cytosolic cargo destined for degradation, which is mediated by the serine/threonine protein kinase ULK1 (unc-51-like kinase 1) (Zachari and Ganley 2017). *ULK1* mRNA was revealed to undergo m^6^A modification in the 3′-UTR, and the m^6^A-marked *ULK1* transcripts can further be targeted for degradation by YTHDF2 (YTH N^6^-methyladenosine RNA binding protein 2). The m^6^A sites on the transcripts were further determined to be the direct substrates of FTO (fat mass and obesity-associated protein), which removes the m^6^A mRNA modification of *ULK1* transcripts, thus promoting autophagy and prolonging the half-life of *ULK1* transcripts (Jin et al. 2018). A further study identified that *Atg5* and *Atg7* transcripts were the targets of YTHDF2, resulting in mRNA degradation and reduction of protein expression, thereby alleviating autophagy (Wang et al. 2020). However, whether autophagy could regulate the mRNA m^6^A modification in feedback and affect the m^6^A-related proteins is not clear. In this study, we demonstrated that PepMV RdRP exploits the autophagy pathway by directly interacting with SlBeclin1 to promote the autophagic degradation of the SlHAKAI protein, which was involved in the m^6^A modification of viral RNA. Thus, this study provides a novel insight into the interplay between autophagy and m^6^A methylation.

Autophagy, as a critical component of plant antiviral innate and adaptive immunity, has been consolidated by numerous studies recently (Nakahara et al. 2012; Hafren et al. 2017, 2018; Haxim et al. 2017; Li et al. 2018a). However, some plant viruses can repress or even manipulate autophagy to counter plant defense. For instance, disruption of autophagy by silencing ATG genes *ATG7* or *ATG8f* or treatment with the autophagy inhibitor 3-MA reduces bamboo mosaic virus (BaMV) accumulation, suggesting that autophagy could play a pro-viral role during BaMV infection (Huang et al. 2019). Rgs-CaM, an endogenous RNA silencing suppressor, was reported to promote geminivirus infection by interacting with the suppressor of gene silencing 3 (SGS3) of RNA silencing to mediate its autophagic degradation in *N. benthamiana* (Li et al. 2017). In another study, the γb protein encoded by barley stripe mosaic virus was revealed to interfere with the interaction of ATG7 and ATG8 in a competitive manner (Yang et al. 2018). Combined with our previous finding (Li et al. 2018a) and this study, we revealed that the PepMV RdRP could become a target of autophagy. However, it could also exploit the autophagy pathway to inhibit m^6^A modification-mediated anti-viral defense by promoting the degradation of SlHAKAI. Therefore, this finding expanded our understanding of the role of autophagy in the mRNA m^6^A modification pathway, indicating its perplexing roles in the context of virus infection.

In summary, our study demonstrates that PepMV RdRP exploits the autophagy pathway by directly interacting with SlBeclin1 to promote the autophagic degradation of the SlHAKAI protein, which is involved in m^6^A modification. Our study highlights the functional importance of the autophagy machinery in regulating m^6^A modification during viral infection. These findings provide insights into the crosstalk among autophagy, m^6^A modification, and viral counter-defense.

## Supplementary Information

Below is the link to the electronic supplementary material.

Supplementary file1 (DOC 2401 KB)

## Data Availability

The data sets generated and analyzed during the current study are available from the corresponding author upon request.
