# Supplementary material for: Pepino mosaic virus antagonizes plant m6A modification by promoting the autophagic degradation of the m6A writer HAKAI
Source: aBIOTECH. 2023 Feb 23;4(2):83–96. doi: 10.1007/s42994-023-00097-6 (PMC10423194; doi:10.1007/s42994-023-00097-6)
Supplement: Supplementary file 1 — Supplementary file1 (DOC 2401 KB) [file 42994_2023_97_MOESM1_ESM.doc]

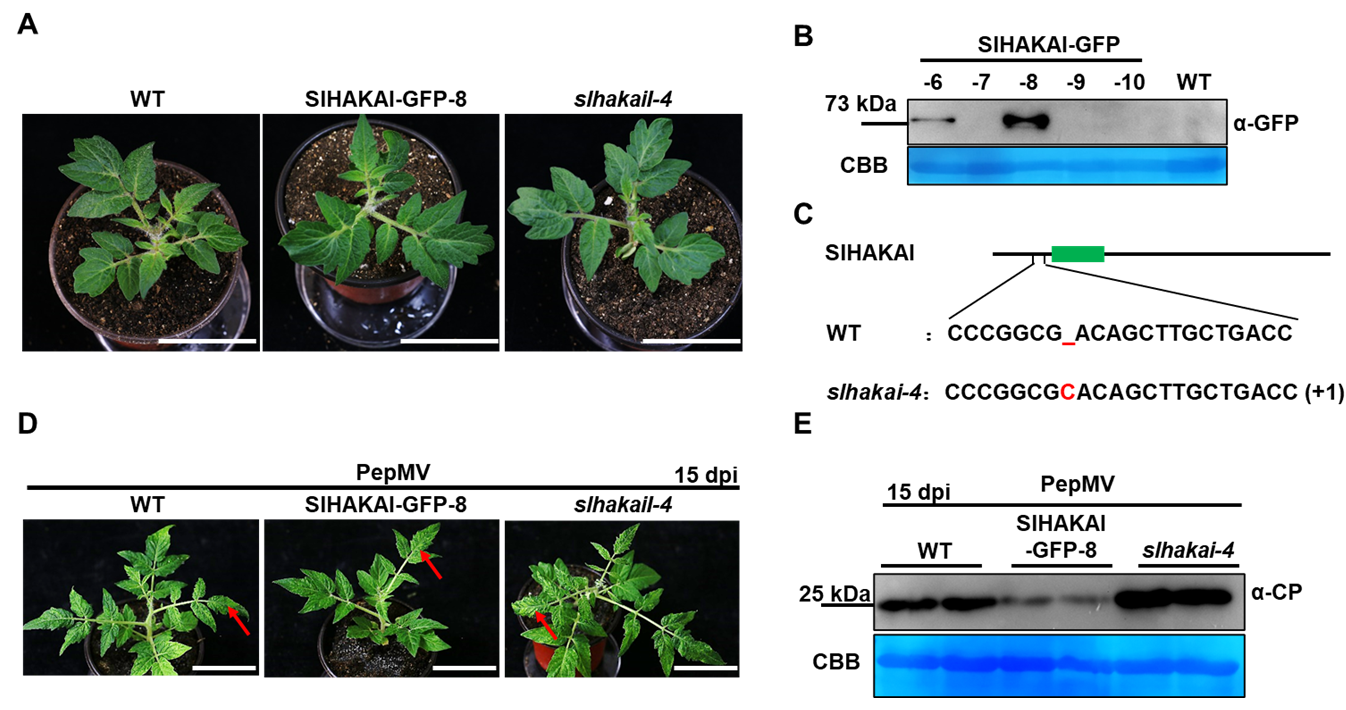


**Fig. S1 SlHAKAI limits PepMV infection in tomato. (A)** The phenotypes of SlHAKAI-overexpression (SlHAKAI-GFP-8) transgene and *SlHAKAI*-knockout (*slhakai-4*) plants in the T2 generation. WT: wild-type (WT). The indicated plants were photographed 30 days after seeding. White bar represents 5 cm. **(B)** Western blot analysis of the SlHAKAI-GFP protein accumulation in SlHAKAI-GFP-overexpression transgenic tomato plants using anti-GFP antibodies. SlHAKAI-GFP-6, 7, 8, 9, 10 represents the 5 individual transgene lines. Coomassie Brilliant Blue R-250 (CBB)-stained Rubisco large subunit was set as a loading control. (**C)** DNA sequencing and sequence alignment were conducted to confirm the mutation in the *SlHAKAI*-knockout (*slhakai-4*) transgene plants*.* **(D)** The pepino mosaic virus (PepMV) caused symptoms in WT, SlHAKAI-GFP-8, and *slhakai-4* tomato plants. The PepMV-inoculated tomato plants were photographed at 15 dpi. The white bar represents 5 cm. **(E)** The accumulation levels of PepMV protein in (D)-indicated plants were determined by Western blotting using anti-PepMV CP antibodies at 15 dpi. CBB-stained Rubisco large subunit was a loading control.


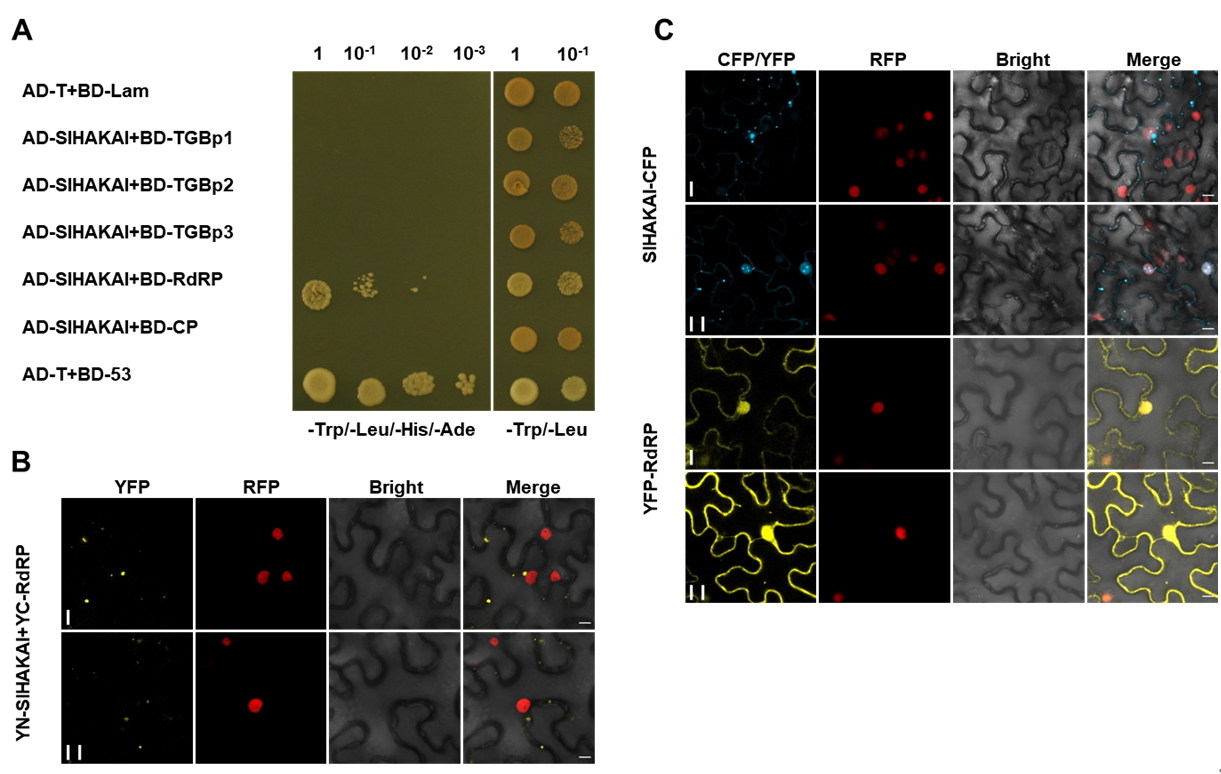


**Fig. S2 SlHAKAI interacts with RdRP.** **(A)** Y2H assays of the interaction between SlHAKAI and PepMV-encoded proteins. Yeast cells co-transformed with AD-T7-T+BD-T7-53 serve as a positive control; yeast cells co-transformed with AD-SlHAKAI and the empty BD, with the empty AD and BD-TGBp1/TGBp2/TGBp3/RdRP/CP, or with AD-T+BD-Lam are negative controls. Y2H Gold yeast cells co-transformed with the indicated plasmids were subjected to 10-fold serial dilutions and plated on synthetic dextrose (SD)/-Trp, -Leu, -His, -Ade or SD/-Trp, -Leu medium to screen for possible interactions at 3 days after transformation. **(B)** BiFC assays between SlHAKAI and RdRP in the leaves of RFP-H2B (red) transgenic *Nicotiana benthamiana* without (I) or with (II) PepMV infection. **(C)** Subcellular localization of SlHAKAI and RdRP without (I) or with (II) PepMV infection.


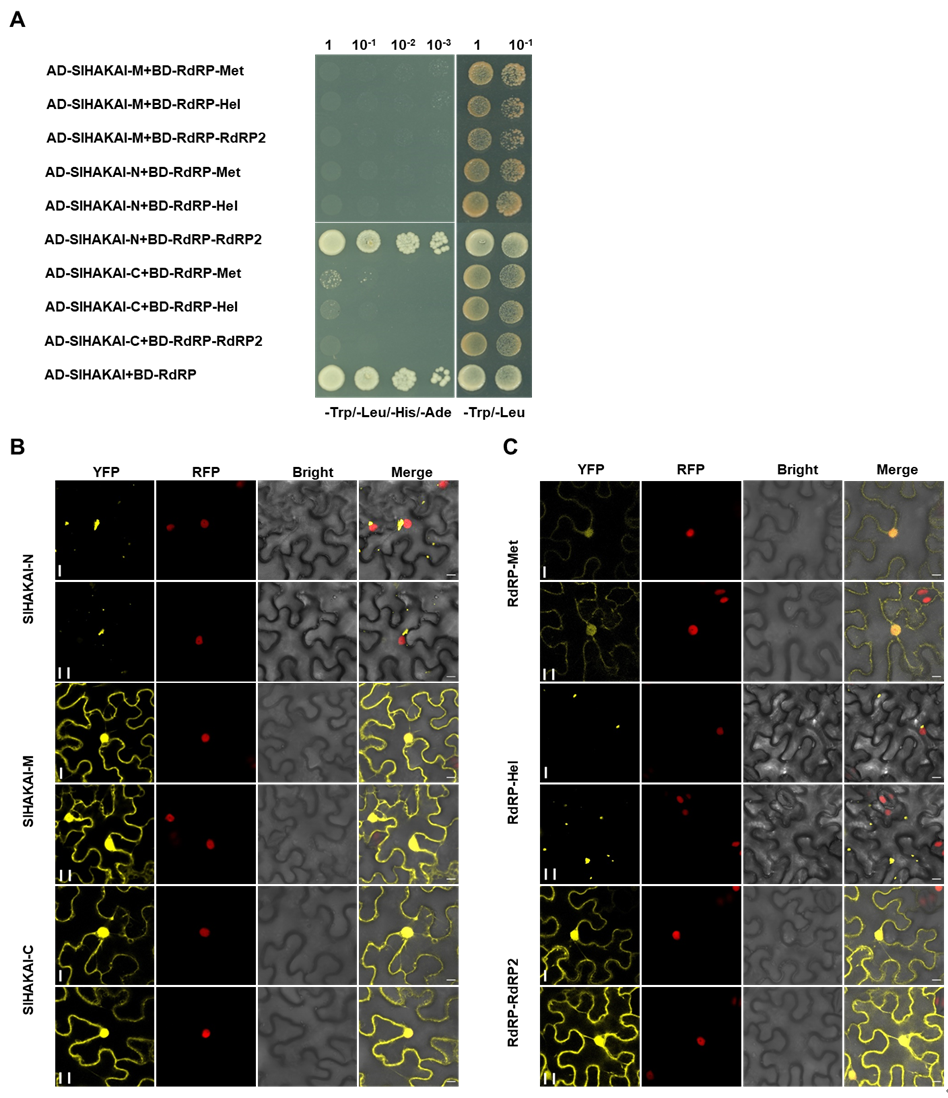


**Fig. S3 Subcellular localization of SlHAKAI, RdRP, and the domains of SlHAKAI and RdRP fused to YFP without or with PepMV infection. (A)** Mapping the interaction domain between SlHAKAI and RdRP by Y2H assays. **(B-C)** Subcellular localization of SlHAKAI-N, M, C and RdRP-Met, Hel, RdRP2 fused with YFP without (I) or with (II) PepMV infection. The YFP signal is shown in yellow. The nuclei of *N. benthamiana* leaf epidermal cells are marked by RFP-H2B (red). These experiments were repeated three times independently. At least 20 cells per sample were observed, and representative results were displayed. Scale bars, 10 µm (B-C).


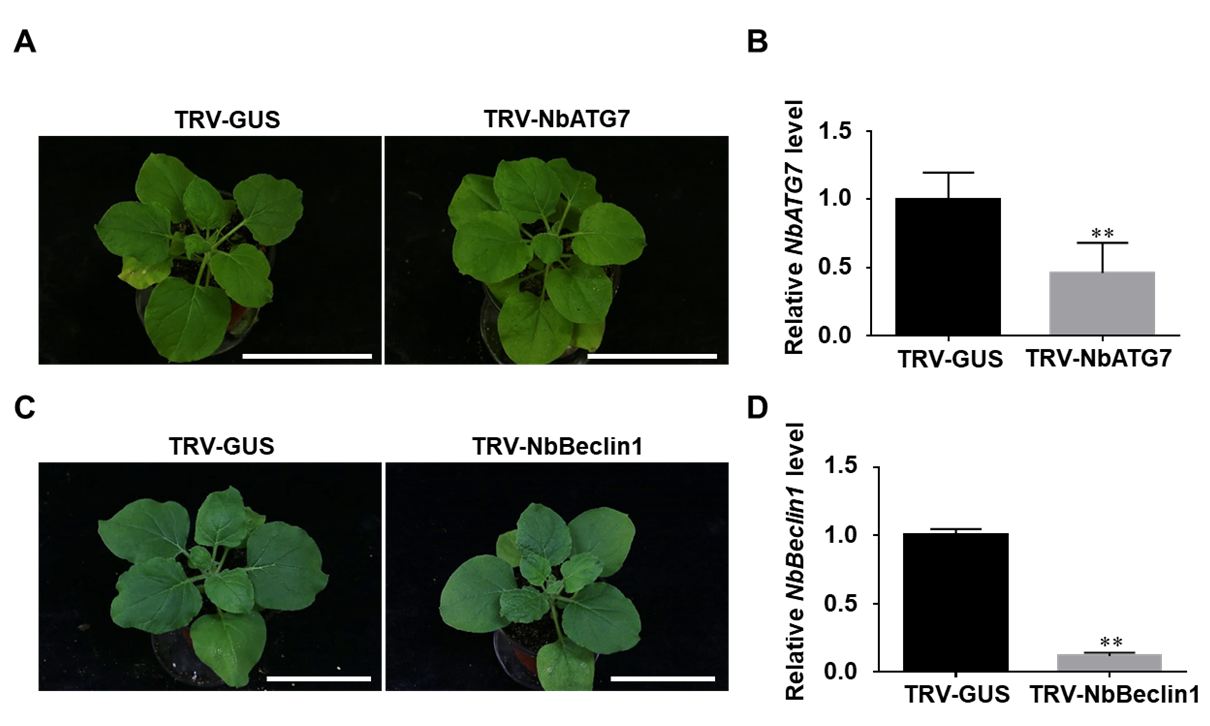


**Fig. S4** **Phenotypes of *NbATG7* or *NbBeclin1*-silenced plants. (A)** Growth phenotypes of the non-silenced (TRV-GUS), *NbATG7*-silenced (TRV-NbATG7) plants at 10 dpi. Bars, 10 cm. Mixed *A. tumefaciens* cultures carrying TRV RNA1+RNA2-GUS (TRV-GUS) and TRV RNA1+RNA2-NbATG7 (TRV-NbATG7) were individually inoculated into *N. benthamiana* leaves. **(B)** Confirmation of the silencing efficiency of *NbATG7* by qRT-PCR. **(C)** Growth phenotypes of the non-silenced (TRV-GUS), *NbBeclin1*-silenced (TRV-NbBeclin1) plants at 10 dpi. Bars, 10 cm. Mixed *A. tumefaciens* cultures carrying TRV-GUS, TRV RNA1+RNA2-NbBeclin1 (TRV-NbBeclin1) were individually inoculated into *N. benthamiana* leaves. **(D)** Confirmation of the silencing efficiency of *NbBeclin1* by qRT-PCR. *NbActin* was regarded as the reference gene to normalize the relative expression, and the value in TRV-GUS-treated plants was set to 1. Values represent the mean ± SD from 3 independent biological samples. Student’s *t*-test was used to analyze each group of data, and double asterisks indicate significant statistical differences (**p<0.01) between the two treatments (B, D).

**Table S1. Primers used in this study (5'–3').**

| SlHAKAI-spacer1-F | TGATTGTTCCGGTAGCCAAGAGCCT |
| --- | --- |
| SlHAKAI-spacer1-R | AAACAGGCTCTTGGCTACCGGAACA |
| SlHAKAI-spacer4-F | TGATTGTCGGTCAGCAAGCTGTCGC |
| SlHAKAI-spacer4-R | AAACGCGACAGCTTGCTGACCGACA |
| SlHAKAI-p1300-F | CGGGGTACCATGCTTCAGATCCGTCTTAG |
| SlHAKAI-p1300-R | CGCGGATCCGTCCTGGTTGCTTCTG |
| SlHAKAI-F | GGGGACAAGTTTGTACAAAAAAGCAGGCTTCATGCTTCAGATCCGTCTTAG |
| SlHAKAI-R | GGGGACCACTTTGTACAAGAAAGCTGGGTCGTCCTGGTTGCTTCTGAAAT |
| q-PepMV-F | GCTTATAGCTCAGATGTTAAGAATAAC |
| q-PepMV-R | GGACAACAACTGTACAGCAGTCATTATTG |
| q-SlHAKAI-F | ACTCTGTCTGCTACCTCTGTGATG |
| q-SlHAKAI-R | CGGGATCCACCCCTTCCAAC |
| q-SlActin-F | AAAGACCAGCTCATCTGTTGAGAAG |
| q-SlActin-R | GTGGTTTCATGAATACCAGCAGC |
| q-NbActin2-F | AAAGACCAGCTCATCCGTGGAGAA |
| q-NbActin2-R | TGTGGTTTCATGAATGCCAGCAGC |
| PepMV-RdRP-F | GGGGACAAGTTTGTACAAAAAAGCAGGCTTCATGTCTCGTGTTAGAAATACTTTGG |
| PepMV-RdRP-R | GGGGACCACTTTGTACAAGAAAGCTGGGTCATGTGTTGCTTTTGGGGG |
| PePMV-Met-F | GGGGACAAGTTTGTACAAAAAAGCAGGCTTCATGTCTCGTGTTAGAAACACTTTAG |
| PePMV-Met-R | GGGGACCACTTTGTACAAGAAAGCTGGGTCTGTGGATATAAGCTGTCTAACTTTTG |
| PePMV-Hel-F | GGGGACAAGTTTGTACAAAAAAGCAGGCTTCATGGTTGTCATACATGGTTGTGG |
| PePMV-Hel-R | GGGGACCACTTTGTACAAGAAAGCTGGGTCATTGATAAAATGGATAGAATCCAC |
| PePMV-RdRP2-F | GGGGACAAGTTTGTACAAAAAAGCAGGCTTCATGAACGGCAACAACTTAGAGAAA |
| PePMV-RdRP2-R | GGGGACCACTTTGTACAAGAAAGCTGGGTCATGTGTTGCTTTTGGGGGGCTC |
| PePMV-RdRP2-dGDD-F | CAGTGAAACAAGTTTACGCTATGGCTCTTGACGGAGTGGTCATG |
| PePMV-RdRP2-dGDD-R | CATGACCACTCCGTCAAGAGCCATAGCGTAAACTTGTTTCACTG |
| SlHAKAI-N-R | GGGGACCACTTTGTACAAGAAAGCTGGGTCAGATTCAAACTCAGTCTCCT |
| SlHAKAI-M-F | GGGGACAAGTTTGTACAAAAAAGCAGGCTTCATGCATATTCATGAGACTC |
| SlHAKAI-M-R | GGGGACCACTTTGTACAAGAAAGCTGGGTCATAAAATGGTTGGGCTCCC |
| SlHAKAI-C-F | GGGGACAAGTTTGTACAAAAAAGCAGGCTTCATGGGTGCTGCTTCAGTTG |
| NbATG7-qPCR-F | AGGTCTCGATGTCTAATCCTCTACGCCAG |
| NbATG7-qPCR-R | AATCAAATCAGACAAATGTCTGCAATCCTG |
| NbBeclin1-qPCR-F | GACCTGCGTAAAGGAGTTTGCTGAC |
| NbBeclin1-qPCR-R | CCAACAAACCAGTAGAGCACCCAC |
